# Comments regarding “Effects of therapeutic interventions on long COVID: a meta-analysis of randomized controlled trials”

**DOI:** 10.1016/j.eclinm.2026.103882

**Published:** 2026-04-23

**Authors:** David Tuller

**Affiliations:** Senior Fellow David Tuller, Center for Global Public Health, School of Public Health, University of California, Berkeley, CA, 94720, USA

A recent article, “Effects of therapeutic interventions on long COVID: a meta-analysis of randomized controlled trials,” reported “high-certainty evidence” that exercise training can improve health and “should be prioritized”.^1^ I would like to raise some concerns regarding this study that may impact the conclusions drawn by the authors.

First, the dozens of trials in the meta-analysis feature heterogeneous definitions of Long COVID. In some cases, it appears that trial participants did not even have Long COVID. For example, a Brazilian study included “subjects with coronavirus disease 2019 in the acute phase”.^2^ Pooling together trials with heterogeneous populations, and including results from participants who do not meet definitions of Long COVID, makes it challenging to interpret the meta-analysis results.

Second, the authors themselves determined that the data used in the meta-analysis must be interpreted with caution. They found that, according to Cochrane's Risk of Bias Tool, only three of the included trials were at “low risk” of bias, with 33–the majority–at “high risk” of bias. Their analysis of quality of evidence for the many outcomes, per the Grading of Recommendations Assessment, Development and Evaluation (GRADE) framework,^3^ yielded similar results, with “27% of the evidence … rated as very low, 57% as low, 11% as moderate, and 5% as high.”

Given these acknowledged limitations, the confident assertion that exercise training is effective and “should be prioritized” is unwarranted, as is the statement that these findings constitute “high-certainty evidence.”

## Contributors

DT conceived of and wrote the letter.

## Declaration of interests

DT's academic position is largely funded by donations, some from people with Long COVID, made directly to the University of California, Berkeley, through the online campus crowdfunding platform.
